# Prevalence and predictors of annual asthma reviews in Scottish primary care data: an observational study

**DOI:** 10.3399/BJGPO.2024.0062

**Published:** 2025-03-12

**Authors:** Holly Tibble, Alexandria Ming Wai Chung

**Affiliations:** 1 Usher Institute, University of Edinburgh, Edinburgh, UK; 2 Asthma UK Centre for Applied Research, Edinburgh, UK; 3 Clinical Infection Research Group, Edinburgh, UK

**Keywords:** asthma, health promotion, large database research, primary health care

## Abstract

**Background:**

People with asthma are recommended to have regular reviews in primary care, with assessment of symptoms, adjustment of treatment and self-management processes, and the delivery of a written action plan for emergencies.

**Aim:**

To investigate the incidence and factors associated with attendance of annual asthma reviews.

**Design & setting:**

This observational study used electronic health records for 49 307 patients in Scotland with asthma between 1 January 2000 and 31 March 2017. The analysis population of 13 726 patients had at least five asthma-related encounters between 2008 and 2016.

**Method:**

Multivariable logistic regression was employed, using linked primary care prescription data and primary care registration demographic data.

**Results:**

There was a median of 381 days between subsequent reviews. Reviews in the index year were strongly associated with reviews in the following year (odds ratio [OR] 1.76, 95% confidence interval [CI] 1.68 to 1.84). In contrast, asthma consultations (excluding reviews) in the index year were associated with lower odds of having a review in the following year (OR 0.48, 95% CI = 0.46 to 0.51). Those aged 18–35 years in the index year or those with missing addresses in the practice registration data were the least likely groups to have an asthma review in the following year.

**Conclusion:**

Reviewing the delivery of asthma care identifies patients who may be slipping through the gaps by receiving only reactive asthma care rather than the structured, preventive care that can be delivered through annual reviews. Understanding the risk factors for not receiving an annual review can be leveraged to create more effective review invitations, such as explaining the specific content of reviews, introducing new contact methods to improve health equity, and reviewing the algorithm used to determine who is invited.

## How this fits in

Clinical guidance stipulates that asthma reviews should be conducted at least once a year, or more often for those with poorly controlled asthma. However, recent studies have reported that insufficient proportions of patients at high risk for serious outcomes had attended a review in the previous year. This study leveraged longitudinal records from 13 726 individuals with asthma, and identified key factors associated with annual review attendance.

## Introduction

Asthma affects ≥300 million people worldwide, including 11.6% of children aged 6–7 years.^1^ In the UK alone, >8 million people (12%) have been diagnosed with asthma, with 160 000 new cases recorded annually.^1^ Asthma accounts for 2%–3% of primary care consultations, and >1000 hospital admissions and 25 deaths per week on average in the UK.^2,3^

National asthma guidelines recommend that patients with asthma should have regular reviews in primary care, with assessment and adjustment of treatment, and self-management processes. The review should culminate in an action plan reminding patients what to do in the event of a decline in symptoms.^4,5^ Action plans should be written or pictorial, and follow evidence-based templates, such as those created by Asthma + Lung UK, which patients take home with them.^6^ Asthma reviews should be conducted at least once a year, or more often for poorly controlled asthma.^7^ The effectiveness of regular reviews on outcomes, such as preventing hospitalisation and accident and emergency (A&E) attendance, has been repeatedly demonstrated across varying populations. In particular, studies have highlighted that personalised guidance has a greater impact on disease prevention than generic documents.^5^

Although those with the most severe asthma presentation are at the highest risk of poor outcomes, evidence suggests not all patients in danger of life-threatening deterioration are aware of their risk or understand how to handle emergency situations.^8^ The 2014 UK National Review of Asthma Deaths only identified evidence of asthma reviews in 23% of those who experienced fatal attacks.^5^

This study had two aims: first, to investigate the proportion of patients with asthma with annual reviews identified in coded primary care data, and consider any changes to incidence and coding practices over time; second, to analyse the associations between having an annual review and patient factors as recorded in primary care.

## Method

### Data

This study uses data from the Asthma Learning Health System (ALHS) study, which had recruited over half a million patients from 75 general practices in Scotland, with primary care records linked to national A&E, hospital, and mortality datasets.^9^

The primary care encounters dataset consisted of 11 766 100 Read code records (for which an encounter might contain multiple codes), for 49 307 unique patients with a diagnosis of asthma, dated between 1 January 2000 and 31 March 2017. The dataset contained a pseudo-anonymised study-patient identifier, the date of the encounter, a unique identification (ID) for the encounter, the Read code (version 2), and three fields for data values, as appropriate (such as numerical value for weight, and unit of weight as a string).

The primary care registration data included demographics for patients, including their age at registration (used to estimate date of birth), sex, the Scottish Index of Multiple Deprivation (SIMD) quintile of their home address (as per the 2012 mapping), and the 6-level Urban Rural Classification (UR6) scale value.

### Analysis population

The analysis population was all individuals identified who had at least five asthma-related encounters between 1 January 2008 and 31 December 2016, of which at least one was after 1 January 2010. The period between 1 January 2008 and 31 December 2009 served as a run-in observation period for patient eligibility criteria, before the study period of 1 January 2010–31 December 2016.

An encounter could either be a consultation or a review. A consultation (defined herein by Read codes in Supplementary Table S1) would typically be an unscheduled encounter, instigated by the patient, to discuss changes in their symptoms or control. In contrast, an asthma review (defined by Read codes in Supplementary Figure S1, along with their prevalence by year) is a scheduled and structured encounter, in which the patient is specifically invited by their GP.

### Analysis plan

Eligible asthma review records were identified within the study period. Review records were excluded if they were duplicates of other records on all fields except the encounter ID. Additionally, if there were <30 days between two encounters marked as annual reviews within a 30-day period, then the second encounter was flagged as a probable follow-up encounter and was removed from subsequent analyses. Summary statistics were calculated for the number of days between subsequent reviews.

Factors associated with incidence of annual reviews were investigated using logistic regression. In this analysis, data from each year for each patient (excluding their final year) was used to predict whether they had an annual review in the following year. As such, a patient with follow-up from 2013–2016 would contribute three ‘index’ years for the analysis (2013, 2014, and 2015), with outcomes taken from the next year (2014, 2015, 2016). Asthma attacks were identified in primary care records from Read codes listed in Supplementary Table S2. Prescriptions were categorised as inhaled corticosteroids (ICS), long-acting beta-2 agonists (LABA), and short-acting beta-2 agonists (SABA). The methods for identifying and classifying medications are described elsewhere.^10^

Factors investigated in univariate analyses were review attendance in previous year (binary), primary care encounters in previous year (none, one, two, or three and more; non-ordinal), and asthma attacks (ever, prior; binary), SABA in previous year (binary), ICS in previous year (none, ICS only, or ICS+LABA; non-ordinal), SIMD (quintiles or unknown; non-ordinal), sex (binary), UR6 (levels 1, 2, 3/4 (combined owing to small numbers), 5, 6, or unknown; non-ordinal), and age category (aged <18 years, 18–35 years, 36–50 years, 51–70 years, ≥71 years; non-ordinal). The same features were used for the multivariate analysis, but with primary care encounters and annual review attendance in the previous year combined into a single categorical feature.

Analysis was conducted in R (version 3.6.1).

## Results

### Study population

Of the 13 726 individuals in the study population (based on history of asthma-related encounters), there was a median of 7.9 years between their first consultation and the end of the observation period (interquartile range 6.3–8.5; range 1.0–9.0). In total, 1241 individuals had no asthma reviews during their follow-up (9.0%), 988 had a single review (7.2%), and 11 497 had multiple reviews (83.8%) (Table 1). There was no clear trend over time of the incidence rate of asthma reviews, with rates stable between 60.2% and 61.9% from 2010–2015, excluding a substantial decrease in 2012 (57.1%) (data not shown).

**Table 1.** Demography of the study population

Characteristic	No reviews, *n* = 1241, *n* (%)	One review, *n* = 988, *n* (%)	Multiple reviews, *n* = 11 497, *n* (%)	All participant*s, n* = 13 726, *n* (%)
**Age in 2010, years**				
<18	82 (6.6)	102 (10.3)	2156 (18.8)	2340 (17.0)
18–35	263 (21.2)	251 (25.4)	1944 (16.9)	2458 (17.9)
36–50	279 (22.5)	265 (26.8)	2801 (24.4)	3345 (24.4)
51–70	363 (29.3)	238 (24.1)	3427 (29.8)	4028 (29.3)
≥71	254 (20.5)	132 (13.4)	1169 (10.2)	1555 (11.3)
**Sex**				
Female	806 (64.9)	655 (66.3)	6883 (59.9)	8344 (60.8)
Male	435 (35.1)	333 (33.7)	4614 (40.1)	5382 (39.2)
**Scottish Index of Multiple Deprivation^a^**				
1 (highest deprivation)	240 (19.3)	225 (22.8)	2478 (21.6)	2943 (21.4)
2	273 (22.0)	200 (20.2)	2375 (20.7)	2848 (20.7)
3	221 (17.8)	172 (17.4)	1987 (17.3)	2380 (17.3)
4	285 (23.0)	219 (22.2)	2509 (21.8)	3013 (22.0)
5 (lowest deprivation)	196 (15.8)	151 (15.3)	1960 (17.0)	2307 (16.8)
Missing	26 (2.1)	21 (2.1)	188 (1.6)	235 (1.7)
**Scottish Urban Rural Classification^a^**				
1 (large urban)	260 (21.0)	266 (26.9)	3257 (28.3)	3783 (27.6)
2 (other urban area)	510 (41.1)	418 (42.3)	4379 (38.1)	5307 (38.7)
3/4 (accessible or remote small towns)	268 (21.6)	139 (14.1)	1666 (14.5)	2073 (15.1)
5 (accessible rural)	148 (11.9)	116 (11.7)	1299 (11.3)	1563 (11.4)
6 (remote rural)	23 (1.9)	19 (1.9)	645 (5.6)	687 (5.0)
Missing	32 (2.6)	30 (3.0)	251 (2.2)	313 (2.3)

^a^Scottish Index of Multiple Deprivation and Urban Rural Classification are both recorded at the patient’s most recent update to their primary care registration details.

Of people aged ≥71 years, 16.3% (*n* = 254/1555) had no asthma reviews recorded, compared with only 9.0% (*n* = 363/4028) in the 51–70 age group. In contrast, only 3.5% of those aged <18 years (*n* = 82/2340) had no asthma reviews (Table 1). In those with at least two reviews (*n* = 11 497), the median number of days between reviews was 381, with an interquartile range of 317–459. A histogram of the gaps between reviews is presented in Supplementary Figure S2.

### Univariate analyses

**Figure 1.**
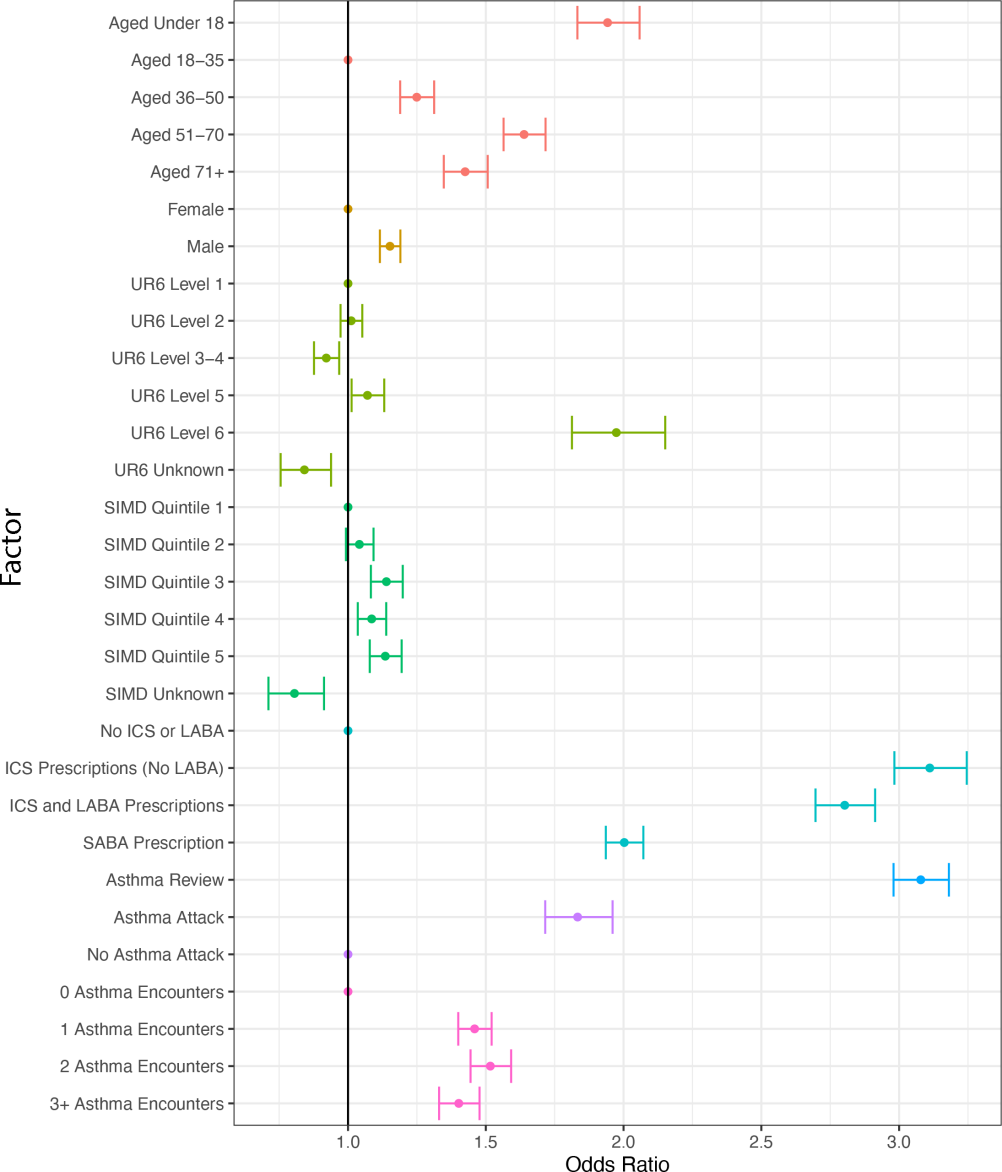
Characteristics in index year and (unadjusted) odds ratio of asthma review in following year. Scottish Index of Multiple Deprivation and Scottish Urban Rural Classification are both recorded at the patient’s most recent update to their primary care registration details. Encounters include both consultations and asthma reviews. ICS = inhaled corticosteroid. LABA = long-acting beta-2 agonist. SABA = short-acting beta-2 agonist. SIMD = Scottish Index of Multiple Deprivation. UR6 = Urban Rural Classification.

Factors associated with having a review in the next year were first analysed using univariate analyses. There were 66 847 person–years in this analysis for all 13 726 individuals in the study. As shown in Figure 1, the most strongly associated (unadjusted) factors in whether an individual had an asthma review in the next year was whether or not they had one in the index year (odds ratio [OR] 3.08, 95% confidence interval [CI] = 2.98 to 3.18) and whether they had been prescribed an ICS in the previous year (ICS alone OR 3.11, 95% CI = 2.98 to 3.24; ICS+LABA OR 2.80, 95% CI = 2.70 to 2.91). However, the association between encounters (either consultations or reviews) in the index year was substantially less strong (asthma encounters, compared with none, in index year ORs 1.40–1.52).

Of the observations (person–years) in which there was an asthma attack recorded in the index year, 74.5% (*n* = 3610/4845) had reviews in the following year, with an OR of 1.83 (95% CI = 1.71 to 1.96) (data not shown).

Those aged 18–35 years in the index year were the least likely to have an asthma review in the following year (Figure 1). There was very little difference between SIMD quintiles, although there was a slight trend with less deprivation being associated with higher odds of review (quintile 5 versus quintile 1 OR 1.13, 95% CI = 1.08 to 1.19). There was no clear trend for the UR6 category, with the exception of level 6 (the most remote, rural patients) having by far the highest odds of a review in the following year (compared with those in level 1 [large urban areas] OR 1.97, 95% CI = 1.81 to 2.15). Unknown SIMD and UR6, resulting from missing address in the primary care registration data, was associated with lower odds of review in the following year (SIMD unknown compared with SIMD quintile 1 OR 0.81, 95% CI = 0.71 to 0.91; UR6 unknown versus UR6 level 1 OR 0.84, 95% CI = 0.76 to 0.94). Figure 1 is also provided in tabular form in Supplementary Table S3.

### Multivariate analyses

In the univariate analyses, the association between reviews in the index year and reviews in the following year was substantially stronger than the association between combined consultations and reviews (‘encounters’) with asthma reviews in the following year. As such, in the multivariate analysis, primary care consultations and asthma reviews in the index year were combined into a single variable (no consultations or reviews, consultations but no review, review but no other consultations, and review and other consultations). In doing so, an interesting association was identified: in those with no review in the index year, having had only a consultation was actually associated with a lower probability of having an asthma review in the following year (OR 0.48, 95% CI = 0.46 to 0.51), compared with having no reviews or consultations in the index year (Table 2). Having had a review was associated strongly with having a review in the following year, but there was little difference regarding whether they had other consultations beside the review in the index year (review but no other consultations OR 1.76, 95% CI = 1.68 to 1.84; review and other consultations OR 1.61, 95% CI = 1.53 to 1.69; 1.61/1.76 = OR 0.91). Besides this, there were no other notable differences between the univariate and multivariate analyses.

**Table 2.** Adjusted odds ratios for characteristics in index year and odds of asthma review in the following year

Characteristic	Adjusted odds ratio	Lower CI	Upper CI
**Year of index observation**	1.080	1.069	1.091
**Age, years**			
<18	1.595	1.499	1.698
18–35	Reference		
36–50	1.158	1.098	1.220
51–70	1.427	1.357	1.500
≥71	1.280	1.204	1.360
**Sex**			
Female	Reference		
Male	1.050	1.014	1.087
**Scottish Urban Rural Classification^a^**			
1 (large urban)	Reference		
2 (other urban area)	1.027	0.983	1.072
3/4 (accessible or remote small towns)	0.933	0.882	0.988
5 (accessible rural)	1.046	0.980	1.116
6 (remote rural)	1.655	1.507	1.818
Missing	1.152	0.915	1.451
**Scottish Index of Multiple Deprivation^a^**			
1 (highest deprivation)	Reference		
2	1.039	0.985	1.095
3	1.092	1.031	1.157
4	1.053	0.996	1.115
5 (lowest deprivation)	1.171	1.105	1.240
Missing	0.790	0.607	1.028
**ICS prescriptions**			
No ICS	Reference		
ICS (no LABA)	2.048	1.951	2.150
ICS+LABA	2.011	1.924	2.102
**SABA prescription**	1.273	1.223	1.325
**Asthma attack**			
No	Reference		
Yes	1.161	1.082	1.246
**Asthma primary care consultations and asthma reviews^b^**			
None	Reference		
Consultation but no review	0.483	0.459	0.509
Review but no other consultations	1.757	1.676	1.842
Review and other consultations	1.605	1.527	1.687

^a^Scottish Index of Multiple Deprivation and Urban Rural Classification are both recorded at the patient’s most recent update to their primary care registration details. ^b^Primary care encounters were specified as consultations and/or asthma reviews in index year, rather than combined as previously into ‘encounters’. ICS = inhaled corticosteroid. LABA = long-acting beta-2 agonist. SABA = short-acting beta-2 agonist.

### Sensitivity analysis

A sensitivity analysis was conducted in which only the Read code '66YJ' ('Asthma annual review') was considered. The percentage of people with no reviews during their follow-up rose only slightly, from 9.0% to 9.8% (*n* = 1346), while the percentage of people with multiple reviews fell from 83.8% to 82.3% (*n* = 11 296). In those with at least two reviews, the median number of days between reviews was 386, with an interquartile range of 336–470. There were no notable differences in the multivariate logistic regression, with results consistent to one decimal place (data not shown).

## Discussion

### Summary

Overall, 9.0% of the study population had no reviews during their follow-up, 7.2% had a single review, and 83.8% had multiple reviews. There was a median of 381 days between subsequent reviews (interquartile range 317–459). In multivariate analyses, reviews in the index year were strongly associated with reviews in the following year. In contrast, asthma consultations (excluding reviews) in the index year were associated with lower odds of having a review in the following year (OR 0.48 in those without review in index year; OR 0.91 in those with a review in the index year). Those aged 18–35 years in the index year were the least likely age group to have an asthma review in the following year (ORs for other age groups were 1.16–1.60 times higher in comparison).

### Strengths and limitations

A major strength of this study is that it was able to leverage electronic health records to extract data from 13 726 individuals. We have identified several strong associations for lower odds of asthma review attendance that can be leveraged to create more effective review invitations; for example, highlighting the differences between the content of routine consultations and structured asthma reviews (added benefit), introducing new contact methods to improve health equity, and reviewing the algorithm used to determine who is invited.

The purpose of primary care records is to support the long-term provision of care. As such, decisions such as suspected diagnosis may be revised based on response to interventions and changes over time. To mitigate misclassification, in this analysis, all individuals were included who had at least five asthma-related consultations or reviews between 1 January 2008 and 31 December 2016, of which at least one was after 1 January 2010. The requirement for a minimum of five encounters was implemented to increase specificity of ascertainment of patients with asthma, and to exclude some individuals for whom this diagnosis was being considered, but ultimately disregarded or superseded.

However, we are unable to ascertain from the available data whether or not an individual was invited to attend an asthma review. In some practices, the list of invited patients is written based on recent history of asthma-related consultations and prescriptions. The latter may partially explain the relationship between ICS (including ICS+LABA combination inhalers) prescriptions in the index year and asthma reviews in the following year. However, it actually adds further intrigue to the finding that in those with no review in the index year, having had encounters in the index year was actually associated with lower odds of having a review in the following year. Further research should investigate whether there is an interaction between prescribing and encounters in the index year with reviews in the following year. However, without the linkage of both prescribing and dispensing data, it is not possible to identify whether a lack of prescriptions is driven by resolution of symptoms (in which case a review would not be required) or poor adherence to medication (in which a review would be highly recommended).^11^ In addition, it is not clear how these results would generalise outside of Scotland, where prescriptions are free, to countries where there may be further barriers to medication dispensing.

Finally, there is no way to appraise the quality of the asthma review, such as whether the review was conducted as the primary reason for the encounter or if it was considered a secondary event during another unscheduled consultation. There is no explicit recording in coded data as to whether an action plan was reviewed and updated. To improve the quality of the outcome ascertainment, and validate the identified associations, data should be extracted from the free-text notes written by the healthcare provider during the encounter. This could also be used to evaluate the clinical code list used, such as whether or not it was appropriate to class Read code '8B3j' ('Asthma medication review', the second most commonly used code in our list) as an asthma review.

### Comparison with existing literature

In 2018, a study of ‘frequent exacerbators’ (≥2 rescue courses of steroids in the past year) in Glasgow, Scotland, identified that only 58% had attended a review in the past year.^12^ In our study, however, 74.5% of observations (person–years) in which there was an asthma attack recorded in the index year had reviews in the following year. This could possibly be explained by variations in the outcome ascertainment, but again further work is required to investigate the relationships between types of medical contacts and annual review attendance to determine whether patients are receiving appropriate care.

These data pertain to healthcare provision in the pre-COVID-19 era. There was mixed evidence as to whether individuals with asthma in the UK were at elevated risk for severe COVID-19 outcomes;^13–16^ however, patients with moderate-to-severe asthma were nonetheless invited for additional vaccinations^17^ and even recommended to shield.^18^ Further work is required to identify any changes in attitudes towards asthma reviews in the wake of the pandemic, and particularly in those who experienced severe symptoms of infection.

### Implications for research and practice

Those without recorded addresses in the primary care registration data had lower odds of asthma reviews, compared with those with known addresses across all SIMD quintiles and UR6 levels. This may represent those who are experiencing homelessness or have no fixed abode (including the travelling community). Further research is required to identify the barriers for these individuals to attend reviews, including how they are invited (typically by postal mail).

In conclusion, this study investigated factors associated with having a recorded annual asthma review in primary care. We observed that asthma consultations (excluding reviews) in the index year were associated with lower odds of having a review in the following year. It is crucial that the delivery of asthma care be reviewed to identify patients who may be slipping through the gaps by receiving only reactive asthma care rather than the structured, preventive care that can be delivered through annual reviews, including crucially the delivery of written emergency asthma action plans.

## Data

The ALHS data are held by the National Services Scotland electronic Data Research and Innovation Service in the National Safe Haven. Restrictions apply to the availability of these data, which were used under license for the current study, and so are not publicly available. Data would be made available from a reasonable request to phs.edris@phs.scot. Analysis was conducted in R (version 3.6.1), and all R scripts are available at: https://github.com/hollytibble/Asthma_Reviews.
